# Influence of Thickener and Mineral Supplementation of Bleaching Gels on Enamel Demineralization

**DOI:** 10.1111/jerd.13495

**Published:** 2025-06-05

**Authors:** Adrielle Caroline Moreira Andrade, Allegra Comba, Nicola Scotti, Leila Es Sebar, Carlos Rocha Gomes Torres

**Affiliations:** ^1^ Department of Restorative Dentistry Institute of Science and Technology, Sao Paulo State University – UNESP Sao Jose dos Campos Brazil; ^2^ Department of Surgical Sciences University of Turin, Dental School, Endodontics and Operative Dentistry Turin Italy; ^3^ Politecnico di Torino, Corso Duca degli Abruzzi Turin Italy

**Keywords:** calcium, demineralization, dental enamel, phosphorus, thickener, tooth bleaching

## Abstract

**Objective:**

This study evaluated the effect of the type of thickener and mineral supplementation of bleaching gels on enamel demineralization.

**Materials and Methods:**

Enamel specimens were divided into five groups according to the type of thickener: CA‐Carbopol, AE‐Aerosil 200, PO‐Poloxamer 407, GG‐Guar gum, and HEC‐Hydroxyethyl cellulose. Each group was subdivided into three subgroups according to the level of mineral supplementation: none, saturation concentration, and maximum addition. The microhardness (KHN), surface roughness (Ra), color change (ΔE00), and mineral content (DD) were measured before and after the treatment, and the changes were calculated. The data was analyzed with two‐way ANOVA and Tukey's test.

**Results:**

ANOVA showed significant differences among the groups (*p* < 0.05). The results of the Tukey test were: %KHN—HEC>GG>CA>PO>AE. %Ra—CA>HEC>PO>GG>AE. DE00—AE>GG>PO>HEC. %DD—HEC>PO>GG>CA>AE. The supplementation reduced the means of %KHN, %Ra, and %DD, without affecting the DE00. The effect of the supplementation level was not statistically significant.

**Conclusion:**

It was concluded that the type of thickener and the use of mineral supplementation significantly influenced the deleterious effects on the enamel.

## 1 Introduction

Dental bleaching is widely used in dental practice because it is effective and low‐cost. Usually, the active ingredient in bleaching gels is either carbamide peroxide or hydrogen peroxide, with the latter being the most commonly used [1]. Dental bleaching promotes chemical alteration of chromophores in the teeth, resulting in a change in color. However, these products can also affect the micromorphology of the enamel surface, raising concerns regarding the safety of the procedure [2, 3]. In fact, mineral loss of enamel may occur during the treatment, reducing microhardness, increasing enamel roughness, and increasing dental erosion susceptibility [4, 5, 6]. Some studies have reported that bleaching gels that maintained a low pH during the application procedure are more aggressive to the enamel. However, bleaching gels with a neutral pH are also capable of causing tooth demineralization [5, 7, 8].

When a bleaching gel is applied to the dental enamel, an exchange of components occurs at the interface between the two materials. If the bleaching gel does not contain calcium and phosphorus in its composition, the tooth releases Ca^2+^_,_ PO_4_^3−^, and OH^−^ ions into the gel until equilibrium is reached, causing tooth demineralization [9]. If the bleaching gel has the addition of calcium (Ca) and phosphorus (P) in its formulation, a supersaturated medium may form in relation to the enamel hydroxyapatite [10]. This way, ions migrate from the bleaching gel to join the enamel, resulting in mineral precipitation. A previous study evaluated the addition of Ca and P salts in a 35% hydrogen peroxide solution and concluded that supplementation above its saturation concentration was able to completely prevent the hardness reduction and roughness increase of the enamel without compromising the bleaching effect [11]. However, for bleaching gels, other studies have shown that supplementation was able to reduce the demineralizing effect but not to completely prevent its occurrence [2, 12, 13]. This suggests that the amount of added salts may have been insufficient to reach saturation concentration, or that additional factors contribute to dental demineralization.

Previous studies showed that even in the absence of peroxide, gels containing certain thickeners were capable of causing tooth demineralization [14, 15]. These studies indicate that the demineralizing effect is not solely attributable to peroxide but also involves other inactive ingredients in the formulation. A thickening agent is defined as a substance capable of increasing the viscosity of a liquid [16]. Regardless of the type used, the thickener can immobilize water and hydrogen peroxide molecules within its structure. Several organic polymers can be used as thickeners, and they can be anionic, cationic, or non‐ionic [17]. Carbomers are the most used anionic thickeners in bleaching gels formulation, with Carbopol 940 and Carbopol 980 being the most well‐known brand examples. They have carboxylic groups connected to the molecular structure of the polymer that, when ionized, exhibit a negative electric charge and result in the expansion of the polymer chain, causing an increase in viscosity. When a gel containing Carbopol is applied to teeth, Ca^2+^ ions released during the ion exchange process bind to the carboxyl groups. As a result, Carbopol acts as a chelator and prevents the reprecipitation of ions as hydroxyapatite, explaining the demineralizing effect [14]. Therefore, a way of controlling the demineralizing effect would be the use of non‐ionic thickeners, such as poloxamer, guar gum, and hydroxyethyl cellulose. Due to their lack of electric charge, they are not expected to exhibit a chelating effect. In addition to organic polymers, inorganic agents such as silica, which also lack electric charge, are commonly used in dentistry for producing acid etching gels [16]. Furthermore, determining the saturation concentration in the bleaching gel may help define the optimal level of supplementation.

Therefore, it can be expected that enough calcium and phosphorus supplementation in association with a less aggressive thickener may be the key to fully controlling the demineralizing effects of the bleaching gel. However, this supplementation must not compromise the bleaching efficacy. Thus, the aim of this study was to evaluate the effect of thickener type and mineral supplementation in bleaching gels on enamel demineralization and bleaching performance. The null hypotheses tested were that neither the thickener type nor the mineral supplementation would affect enamel demineralization or the bleaching effect.

## 2 Materials and Methods

### 2.1 Preparation of Experimental Bleaching Gels

Five experimental bleaching gels were formulated using the thickeners listed in Table 1. A 60% hydrogen peroxide solution (Kolovec, Sao Paulo, SP, Brazil) was used to achieve a final concentration of 35%. The pH was adjusted using triethanolamine (Volp, Osasco, SP, Brazil). Mixing was performed using a dual asymmetric centrifugal mixer (Speed Mixer, FlackTek, Landrum, SC, USA), with a rotation speed of 3500 rpm for 1 min. The amount of thickener in each formulation was adjusted to reach the final viscosity of 200.00cP, consistent with most commercially available products [18]. Viscosity was measured using a rotational viscometer (DV2T, Brookfield, Middleborough, MA, USA) with a Helipath Stand system (Brookfield, Middleborough, MA, USA). The pH measurements were performed using a small‐volume combination electrode (HI1083B, Hanna Instruments, Woonsocket, RI, USA) connected to a benchtop pH meter (DM‐20, Digimed, Sao Paulo, SP, Brazil). The hydrogen peroxide concentration in the gels was determined using a potentiometric titrator (HI902C1‐02, Hanna, Woonsocket, RI, USA). For that, a small sample of each gel was diluted in 20 mL of 1 M H_2_SO_4_ solution inside a Becker flask and kept under constant stirring. Aliquots of 0.1 N KMnO_4_ were automatically added by the machine, and the changes in the redox potential were detected by an electrode (3131B, Hanna, Woonsocket, RI, USA). The hydrogen peroxide concentration was calculated according to the amount of KMnO_4_ required to reach the equivalence point of the reaction.

**TABLE 1:** Thickeners used in the bleaching gels formulation.

Thickeners	Type	Electric charge	CAS	Manufacturer
Carbopol 980	Synthetic polymer (Carbomer)	Anionic	9003‐01‐4	Lubrizol, Wickliffe, OH, USA
Aerosil 200	Particulate colloid, inorganic	Non‐ionic	112945‐5	Evonik, Essen, North Rhine‐Westphalia, Germany
Poloxamer 407	Synthetic polymer	Non‐ionic	9003‐11‐6	Merck, Darmstadt, Hessen, Germany
Guar gum	Natural polymer	Non‐ionic	9000‐30‐0	Dinamica, Indaiatuba, SP, Brazil
Hydroxyethyl cellulose	Semi‐synthetic	Non‐ionic	9004‐62‐0	Maian, Itapevi, SP, Brazil

### 2.2 Determination of the Calcium and Phosphate Saturation Concentration

To determine the saturation concentration of calcium and phosphorus for each experimental bleaching gel, enamel powder was obtained from the crowns of 10 bovine incisors. The teeth were sectioned on the cemento‐enamel junction, and the roots were discarded. Dentin was removed from the crowns using a large round diamond bur. The crowns were cleaned to remove residues and stains, washed, completely dried, and ground using a metal mortar and pestle. The powder was sieved to separate particles between 75 and 150 μm [19].

Five grams of dental enamel powder were mixed with 10 g of each experimental bleaching gel, mixed, and taken to a stove at 37°C for 72 h, allowing the gels to interact with the tooth enamel until reaching an equilibrium on the ionic exchange and saturation concentration. After this time, the enamel particles were separated by centrifugation, and the calcium and phosphorus concentrations in the eluted solution for each bleaching gel were determined by Energy Dispersive X‐ray Fluorescence Spectrometer (EDX—8000, Shimadzu, Kyoto, Kansai, Japan). This method identifies and quantifies the x‐rays generated by the elements inside the sample when hit by an electron beam. Calibration curves were prepared using calcium and phosphate standards (Sigma Aldrich, San Luis, MI, USA), from 0.2 to 1 ppm, which were placed into the liquid sample holder and analyzed. After that, each eluted solution was taken to the holder and the reading was performed. The unknown concentration in ppm was determined by linear regression equations from the calibration procedure.

### 2.3 Determination of the Solubility Limit of Calcium and Phosphorus Salts

To identify the most soluble salts for supplementation, 11 types of calcium salts and five types of phosphorus salts were tested to determine their solubility limit in a peroxide solution. For that, 100 g of each salt was mixed with 100 g of 35% hydrogen peroxide solution for 2 min. Undissolved salts were filtered under vacuum, and the solubility limit was calculated. The molar concentration of calcium or phosphorus in these solutions was determined. The calcium acetate and sodium phosphate dibasic dodecahydrate salts showed the highest molar concentration of the respective elements and were used in the next part of the experiment.

### 2.4 Preparation of Bleaching Gels With Mineral Supplementation

Each experimental bleaching gel received two different levels of mineral supplementation, which were “saturation concentration” and the “maximum addition”. For the first level, the salts were added to the gels until reaching the same level of saturation concentration in ppm determined during the analysis with enamel powder. For the second level, the maximum supplementation possible was added based on the Ca and P molar concentration obtained during the solubility limit analysis.

### 2.5 Specimen Preparation

A total of 360 cylindrical specimens with a diameter of 4 mm were obtained from the buccal surface of bovine incisors. Enamel and dentin were standardized at 1 mm each in order to simulate the thickness of these tissues in a central incisor [20]. Specimens were embedded in bis‐acryl resin composite (Structur 3, Voco, Cuxhaven, LS, Germany) shade B1 using a silicone mold. The specimens had a lateral groove necessary for repositioning in the reading devices. The enamel surface was flattened and polished with SiC abrasive discs (P1200, P2400, P4000, Struers, Copenhagen, Zealand, Denmark) and two diamond suspensions (DiaPro Dac 3 and DiaPro Nap 1, Struers, Copenhagen, Zealand, Denmark) with abrasive cloth, using an automatic polishing machine (Tegramin 25, Struers, Copenhagen, Zealand, Denmark). Specimens were stored in 2 mL of artificial saliva composed of 2.1 mmol/L hydrogen carbonate, 16.1 mmol/L potassium, 14.5 mmol/L sodium, 2.6 mmol/L hydrogen phosphate, 0.8 mmol/L boric acid, 0.7 mmol/L calcium, 0.4 mmol/L thiocyanate, and 0.2 mmol/L magnesium (pH of 7.4) [21].

### 2.6 Baseline Readings

Baseline enamel microhardness of all specimens was measured using a microhardness tester with a Knoop indenter (FM‐700, Future‐Tech, Tokyo, Kanto, Japan). Three indentations were made per specimen, with a 50 g load for a 10 s dwell time, and the KHN mean was calculated. Surface roughness was measured with a contact profilometer (MarSurf GD 25, Mahr, Goettingen, LS, Germany). Three readings were performed per specimen, with a cut‐off of 0.250 mm, and the mean Ra values were calculated. Color measurements were taken with a reflectance colorimetric spectrophotometer (CM 2600d, Konica Minolta, Osaka, Kansai, Japan). The instrument was set for D65 standard illuminant, 100% UV included, 2° for observer angle, specular component included (SCI), and small area view (SAV). Reflectance data were converted into L* a* b* chromatic coordinates using Spectramagic NX software (Konica Minolta, Osaka, Kansai, Japan). Three readings were made per specimen, and the mean calculated.

### 2.7 Specimens' Stratification

The specimens were stratified into different groups according to the microhardness values. To certify the similarity between the groups after the initial distribution, one‐way analysis of variance (ANOVA) was used for microhardness, roughness, and L*, a*, and b* values.

### 2.8 Groups/Subgroups Assignment

Three hundred specimens were divided into five experimental groups based on thickener type agent, as shown in Table 1. Each experimental group was further subdivided into three subgroups according to mineral supplementation (*n* = 20). The first subgroup did not receive any mineral supplementation. In the second subgroup, the Ca and P salts were added according to the saturation concentration (SC) calculated, while for the third subgroup, the maximum addition (MA) of Ca and P was performed according to the solubility limit.

The remaining 60 specimens were divided into three control groups. The negative control was treated with ultrapure water instead of a bleaching agent. For the positive control, the bleaching treatment was performed with a 35% hydrogen peroxide solution. For the commercial positive control, the bleaching was performed using 35% hydrogen peroxide gel (Whiteness HP, FGM, Joinville, Santa Catarina, Brazil). The application time was the same for all experimental and control groups. The baseline and final pH for all groups can be seen in Table 5.

### 2.9 Bleaching Procedure and Final Readings

Specimens were placed in silicone molds and treated with 50 mg of bleaching gel from each corresponding group They were kept at 37°C for 45 min. After this time, the gel was collected, the surface was washed with air‐water spray, and dried with gauze. Post‐treatment microhardness and roughness were measured, and specimens were stored in Eppendorf tubes containing 2 mL of artificial saliva, with daily changes for 14 days, to allow rehydration and release of the hydrogen peroxide from inside the tooth structure. Then, the final color reading was carried out.

Percentage of hardness (%KHN) and roughness (%Ra) modification were calculated considering the final and baseline readings, using the following formula:

$$\%\text{modification}=\left(\left(\text{final}\times 100\right)/\text{baseline}\right)-100.$$

Color change was calculated according to the CIEDE 2000 formula (ΔE00) following the International Commission on Illumination recommendations [22]. The final hydrogen peroxide concentration and the pH of the gel collected from the specimens was measured. The percentage of hydrogen peroxide concentration change was calculated.

### 2.10 Raman Spectroscopy

The effect of the bleaching treatment on the enamel structure was evaluated by Raman Spectroscopy. New specimens for all groups were prepared in the same way as already described (*n* = 10) and the same treatments were performed. Surface measurements were performed before and after bleaching treatment, and the degree of demineralization (%DD) was calculated.

A modular spectrometer (I‐Raman ex, BWTEK, Plainsboro, NJ, USA) with a monochromatic excitation laser (wavelength 1064 nm) was employed. It is coupled with a spectrometer BTC284N (BWTEK, Plainsboro, NJ, USA) with a measurement range of 100–2500 cm^−1^ and a resolution of 10.56 cm^−1^, paired with a CCD sensor. The measurements were performed using a 200 mW power laser, with 40 s of integration time and 12 repetitions. These parameters were defined to maximize the signal/noise ratio and avoid alteration of samples. The whole set was connected to a portable compact Raman microscope (BAC151, BWTEK, Plainsboro, NJ, USA) with an 80x microscopic lens (spot diameter of about 20 μm).

The spectra were processed using a script written in the Python programming language. In particular, the baseline was removed through the asymmetric least squares smoothing, and the Savitzky–Golay filter was applied with a window length of 15 cm^−1^. A standard normal variate transformation (SVN) was applied, and the mean value of the spectra was subtracted from each signal intensity, which is then divided by its standard deviation [23]. Finally, the spectra were narrowed to the region of interest containing the phosphate peak at 960 cm^−1^, relevant to the %DD calculation, i.e., in the range between 800 cm^−1^ and 1200 cm^−1^.

The spectra were further processed with a script developed in Python to perform a deconvolution using a Lorentzian function to extract the amplitude values of the 960 cm^−1^ peak. The DD% was calculated as follows:

$$\mathrm{DD}\%=\left(1-\frac{I_{960}\;\mathrm{PB}}{I_{960}\;\mathrm{BB}}\right)*100$$

where *I*_960_ BB and *I*_960_ PB are the intensities of the vibration modes at 960 cm^−1^ before and after bleaching, respectively [24].

### 2.11 Statistical Analysis

Data normality and homoscedasticity were assessed using Shapiro‐Wilk and Levene tests. A two‐way analysis of variance (ANOVA) test (type of thickener × mineral supplementation) was performed, followed by Tukey's test. The comparisons between the experimental groups and the control groups were carried out using the Dunnett's test. For all analyses, a significance level of 5% was adopted.

## 3 Results

The saturation concentration (SC) values for calcium (Ca) and phosphorus (P) in the 35% hydrogen peroxide gels containing the different thickeners are shown in Table 2. It can be seen that Carbopol removed more minerals from the enamel to reach saturation than any other thickener tested, followed by Hidroxietil cellulose and Guar gum. Aerosil and Poloxamer were the less aggressive ones.

**TABLE 2:** Ca and P saturation concentration in bleaching gels.

Thickeners	Ca (ppm)	P (ppm)
Carbopol 980	595.6	414.3
Aerosil 200	22.4	59.5
Poloxamer 407	29.5	95.2
Guar gum	174.7	131.9
Hydroxyethyl cellulose	236.4	95.2

The two‐way ANOVA for %KHN and %Ra revealed significant differences in effects for both thickener type (*p* = 0.001) and mineral supplementation (*p* = 0.001), as well as their interaction (*p* = 0.001). For color change, significant differences were observed for the thickening factor (*p* = 0.001) but not for mineral supplementation (*p* = 0.841) or the interaction between them (*p* = 0.345). For %DD, significant differences were observed for the factors type of thickeners (*p* = 0.001) and mineral supplementation (*p* = 0.001), as well as for the interaction between them (*p* = 0.001). The results of Tukey's test for the interactions are shown in Table 3.

**TABLE 3:** Means (SD) of %KHN, %Ra, ΔE00, %DD and results of Tukey test for the interaction.

Thickener	Suppl.	%KHN	%Ra	ΔE00	%DD
Carbopol 980	None	−10.06 (0.97)^cd^	139.45 (34.72)^e^	3.29 (0.99)^bcd^	0.57 (2.48)^ab^
	SC	−6.37 (2.97)^e^	16.97 (14.41)^c^	3.82 (1.31)^cd^	−0.21 (1.19)^a^
	MA	−6.06 (2.34)^ef^	10.04 (5.35)^bc^	4.08 (1.47)^cd^	−0.36 (0.81)^a^
Aerosil 200	None	−2.09 (1.02)^gh^	32.39 (21.50)^d^	4.09 (1.37)^cd^	1.81 (3.84)^ab^
	SC	0.90 (0.98)^hi^	−7.85 (5.73)^a^	4.22 (1.32)^cd^	0.15 (1.60)^ab^
	MA	2.90 (1.72)^i^	−8.94 (5.59)^a^	4.39 (1.50)^d^	0.30 (0.17)^ab^
Poloxamer 407	None	−10.46 (3.07)^cd^	39.36 (18.25)^d^	2.26 (0.92)^ab^	14.10 (1.46)^c^
	SC	−2.83 (3.00)^gh^	4.50 (9.33)^abc^	2.27 (0.92)^ab^	−0.79 (1.59)^a^
	MA	−0.51 (1.02)^fg^	4.00 (7.57)^abc^	2.13 (0.74)^a^	−0.30 (3.27)^a^
Guar gum	None	−16.62 (2.39)^b^	33.36 (7.46)^d^	2.62 (0.69)^ab^	0.50 (1.20)^ab^
	SC	−8.13 (2.39)^de^	−0.92 (5.00)^ab^	2.04 (0.54)^a^	1.70 (3.16)^ab^
	MA	−6.16 (4.95)^ef^	−3.31 (3.41)^ab^	2.11 (0.57)^a^	0.15 (0.16)^ab^
Hydroxyethyl cellulose	None	−22.49 (2.64)^a^	153.55 (8.38)^e^	3.35 (0.83)^bcd^	19.63 (1.78)^d^
	SC	−13.05 (4.02)^c^	3.47 (7.75)^abc^	3.11 (1.03)^abc^	1.70 (2.13)^ab^
	MA	−10.66 (6.72)^cd^	3.71 (10.41)^abc^	2.48 (0.75)^ab^	3.13 (2.59)^b^

*Note:* Means followed by the same letter within a column are not significantly different according to the Tukey test.

Abbreviations: %DD, percentage of demineralization; ΔE00, color difference; %KHN, percentage of microhardness change; MA, maximum addition; %Ra, percentage of microhardness change; SC, saturation concentration.

For microhardness changes, the hydroxyethyl cellulose thickener without mineral addition produced the greatest reduction for all thickeners tested, while Aerosil was the less aggressive. The mineral supplementation reduced the negative effect for all thickeners, and the maximum addition was the most effective. The maximum addition for Aerosil increased the hardness in relation to the baseline.

For roughness change it was observed that hydroxyethyl cellulose and Carbopol without mineral supplementation were the most aggressive, while the other thickeners were similar. The mineral supplementation reduced the effect, without significant differences in relation to the amount added. For Aerosil 200 and Guar Gum the roughness after the treatment was smaller than at the baseline.

For color change, the gels with Poloxamer and Guar Gum, the groups without supplementation resulted in smaller bleaching in relation to Carbopol and Aerosil. The mineral addition did not increase the bleaching for the same thickener.

For %DD the group hydroxyethyl cellulose without supplementation showed the highest values. The mineral supplementation similarly reduced the demineralization effect despite the level of addition.

The results for Dunnett test are presented in Table 4. For microhardness, Aerosil alone or supplemented groups did not differ significantly from the negative control group. Only the Guar gum and hydroxyethyl cellulose were statistically similar to the positive control. The others showed lower means of microhardness reduction. In relation to the commercial control group, the hydroxyethyl cellulose supplemented, guar gum, Poloxamer, and Carbopol groups did not show significant differences. For roughness, the Dunnett test showed that mainly non‐supplemented groups showed differences in relation to the negative control. All groups showed significant differences for the positive and commercial control groups. For color change, the Dunnett test showed that all groups differed from the negative control group. Most groups also differed significantly from the positive and commercial control groups. For %DD, the Dunnett test showed that only poloxamer and hydroxyethyl cellulose groups showed significant differences in relation to the negative control.

**TABLE 4:** Results of the Dunnett test for %KHN, %Ra, ΔE00 and %DD.

		Negative control				Positive control				Commercial control			
		%KHN	%Ra	ΔE00	%DD	%KHN	%Ra	ΔE00	%DD	%KHN	%Ra	ΔE00	%DD
**T**	**S**	0.05 (0.27)	3.31 (4.45)	0.41 (0.24)	0.009 (0.99)	−15.05 (2.75)	222.91 (44.93)	3.01 (0.24)	0.44 (0.57)	−13.83 (3.95)	225.09 (50.22)	3.47 (0.91)	0.57 (0.58)
Carbopol	None	0.0001*	0.0001*	0.0001*	0.9997	0.0001*	0.0001*	0.9915	1.0000	0.0030*	0.0001*	0.9999	1.0000
Carbopol	SC	0.0001*	0.0114*	0.0001*	1.0000	0.0001*	0.0001*	0.1300	0.9927	0.0001*	0.0001*	0.9584	0.9964
Carbopol	Max	0.0001*	0,6190	0.0001*	0.9999	0.0001*	0.0001*	0.0152*	0.9683	0.0001*	0.0001*	0.4465	0.9824
A200	None	0.2143	0.2143	0.0001*	0.3548	0.0001*	0.0001*	0.0127*	0.8151	0.0001*	0.0001*	0.4086	0.8742
A200	SC	0.9898	0.0001*	0.0001*	1.0000	0.0001*	0.0001*	0.0033*	0.9999	0.0001*	0.0001*	0.1963	1.0000
A200	Max	0.0346	0.0395*	0.0001*	1.0000	0.0001*	0.0001*	0.0001*	1.0000	0.0001*	0.0001*	0.0576*	0.0001*
Polox.	None	0.0001*	0.0001*	0.0001*	0.0001*	0.0001*	0.0001*	0.1952	0.0001*	0.0118*	0.0001*	0.0035*	0.0001*
Polox.	SC	0.0321*	1.0000	0.0001*	0.9999	0.0001*	0.0001*	0.2094	0.7260	0.0001*	0.0001*	0.0040*	0.8017
Polox.	Max	0.0999*	1.0000	0.0001*	0.9931	0.0001*	0.0001*	0.0779*	0.9819	0.0001*	0.0001*	0.0001*	0.9921
Guar G.	None	0.0001*	0.0001*	0.0001*	0.9999	0.06300	0.0001*	0.9113	1.0000	0.0628*	0.0001*	0.0993*	1.0000
Guar G.	SC	0.0001*	0.9696	0.0001*	0.4342	0.0001*	0.0001*	0.0554*	0.8878	0.0001*	0.0001*	0.0001*	0.9286
Guar G.	Max	0.0001*	0.6381	0.0001*	1.0000	0.0001*	0.0001*	0.0679*	0.9999	0.0001*	0.0001*	0.0001*	0.9999
Hydroxy.	None	0.0001*	0.0001*	0.0001*	0.0001*	0.0001*	0.0001*	0.9641	0.0001*	0.0001*	0.0001*	1.0000	0.0001*
Hydroxy.	SC	0.0001*	1.0000	0.0001*	0.4360	0.3231	0.0001*	1.0000	0.8891	0.9975	0.0001*	0.9454	0.9296
Hydroxy.	Max	0.0001*	1.0000	0.0001*	0.0104*	0.0001*	0.0001*	0.6342	0.0650*	0.0218*	0.0001*	0.0336*	0.1058

Abbreviations: %DD, percentage of demineralization; ΔE00, color difference; %KHN, percentage of microhardness change; MA, maximum addition; %Ra, percentage of microhardness change; SC, saturation concentration; S, Supplementation; T, Thickeners.

* Significant differences.

The hydrogen peroxide concentrations for all tested gels before and after bleaching can be seen in Table 5. The concentrations reduction after 45 min varied substantially among the groups, with the greatest change seen in Carbopol and hydroxyethyl cellulose with the maximum supplementation and the smaller change in the Aerosil 200 group. The pH values changed after the treatments for all groups, and for some thickeners the supplementation reduced the pH changes.

**TABLE 5:** Means (SD) of baseline and final hydrogen peroxide concentrations and pH for all groups and the percentage of change.

Groups		bC	fC	%HP	bpH	fpH
Negative control		—	—	—	7.01	7.01
Positive control		35.78 (0.21)	32.08 (0.08)	10.34	7.01	6.9
Commercial control		34.91 (0.40)	27.51 (0.64)	21.19	6.49	5.29
Carbopol 980	None	35.46 (0.39)	26.51 (0.37)	25.23	7.01	5.68
	SC	35.85 (0.12)	28.37 (0.45)	20.86	7.2	6.8
	Max	35.92 (0.17)	22.32 (0.17)	37.86	7.2	6.4
Aerosil 200	None	35.05 (0.04)	34.54 (0.40)	1.46	7.02	5.8
	SC	35.05 (0.04)	34.21 (0.17)	2.40	7.01	6.2
	Max	35.64 (0.43)	24.34 (0.09)	31.71	7.1	6.43
Poloxamer 407	None	35.53 (0.44)	32.32 (0.17)	9.04	7.1	6.1
	SC	35.77 (0.23)	30.69 (0.37)	14.21	7.2	6.6
	Max	35.69 (0.54)	31.11 (0.10)	12.84	7.1	6.5
Guar gum	None	35.26 (0.35)	30.50 (0.02)	13.50	7.01	5.94
	SC	35.33 (0.18)	32.50 (0.02)	8.02	7	6.3
	Max	35.32 (0.18)	32.24 (0.11)	8.73	7.2	6.7
Hydroxyethyl cellulose	None	35.35 (0.20)	28.86 (0.31)	18.36	7	5.68
	SC	35.61 (0.08)	28.47 (0.39)	20.06	7.2	6.1
	Max	36.99 (0.11)	25.27 (0.43)	29.81	7.1	6.3

Abbreviations: bC, baseline peroxide concentration; bpH, baseline pH; fC, final concentration; fpH, final pH; %HP, percentage of hydrogen peroxide concentration reduction.

## 4 Discussion

The results of this study demonstrated that the thickener type significantly influenced enamel microhardness, surface roughness, color change, and demineralization levels, thereby rejecting the null hypothesis (Table 3). The mineral supplementation affected the microhardness, roughness, and demineralization, but not the bleaching effect, partially rejecting the null hypothesis. The negative control did not show significant changes for all parameters analyzed, indicating that the methods used were adequate (Table 4).

Dental bleaching is one of the most demanded aesthetic procedures by patients due to the impact that dental aesthetics has on people's lives [25]. Trans and postoperative sensitivity and gingival irritation are the most common adverse effects associated with the tooth bleaching procedure [26]. However, certain microscopic changes in enamel structure are also very relevant, as they can cause a drop in microhardness and an increase in surface roughness as a result of a demineralization process generated by the bleaching agent [13, 25].

Hydroxyethyl cellulose is widely used in cosmetics and the pharmaceutical industry. Being a cellulose‐based polymer with a non‐ionic nature, it can be associated with different active principles and shows easy dispersion [27]. It was found that the gel prepared with this thickener without mineral supplementation produced the greatest reduction in microhardness and one of the greatest increases in surface roughness, which is correlated with the higher values of enamel demineralization detected by Raman Spectroscopy (Table 3). The pH was also reduced substantially during the treatment (Table 5), from 7 to 5.68, which may also have contributed to the demineralizing effect [13]. The bleaching effect observed was similar to Carbopol groups and to the positive and commercial controls, although with a smaller peroxide decomposition rate. Despite the amount added, mineral supplementation reduced the negative effects on the microhardness and roughness, without significantly affecting the bleaching effect (Table 3), even though the decomposition rate increased. The pH reduction after the bleaching was smaller, which may be correlated with the smaller demineralization (Table 5).

The Guar Gum thickener is considered an anionic natural polymer, composed of sugars, galactose, and mannose. After hydroxyethyl cellulose, it was the thickener that caused most changes in microhardness and roughness, although it was not detected by Raman. It was also observed that there was a substantial reduction of the pH (Table 5), which may have contributed to demineralization, with levels of hardness reduction similar to the positive control without thickener (Table 4). The bleaching effect was smaller than the commercial control, reducing its clinical application (Table 4). To reach the desired viscosity level, it was the thickener that required the smallest amount when compared to the other groups and showed a small decrease of hydrogen peroxide concentration in relation to hydroxyethyl cellulose. These findings suggest that the extent of mineral loss may be more closely related to the chemical behavior of hydrogen peroxide and the final pH of the gel than to the absolute amount of thickener used in the formulation [28]. The mineral supplementation reduced the negative effects on the enamel, despite the level of addition, although it did not affect the bleaching effect (Table 3).

The Poloxamer 407 thickener presented the second lowest microhardness change, behind only that of Aerosol 200. For roughness increase, together with Carbopol, it presented the highest values, which may be caused by the pH drop. For the preparation of the bleaching gel, a concentration of 46% was required, being the one for which more thickening agent was required for the gel formation. This may be responsible for the smaller bleaching effect of this material in relation to the other gels with different thickeners and to the positive and commercial control groups (Table 4). In addition, a small peroxide degradation (Table 5) was observed that may be correlated to a reduced release of the peroxide molecules from the gel to interact with the tooth structure. The mineral supplementation reduced the negative effects on the tooth structure without affecting the bleaching efficacy.

Although Carbopol 980 is the most used thickener for dental bleaching formulation, it has chelating properties and is anionic [29]. Thus, when the gel is applied to the tooth, the thickener molecules bind to the calcium ions released from the hydroxyapatite, causing tooth demineralization (Table 3) [19, 30]. Previous studies have reported a decrease in microhardness after bleaching with Carbopol containing gels [31, 32], corroborating our study. This group showed the greatest degradation of hydrogen peroxide in relation to the non‐mineral supplemented groups, with the greatest decrease in concentration of 25.23%, besides a substantial pH reduction (Table 5). The largest amount of triethanolamine was necessary to adjust the pH to 7 in relation to the other thickeners. This may have positively affected the bleaching effect, which was similar to commercial control. The mineral addition also reduced the negative effects, despite the amount added, without affecting the bleaching efficacy.

The bleaching gel prepared with Aerosil 200 showed the smallest effect on microhardness in relation to all the other thickeners tested (Table 3), being even smaller than the positive control without thickener and the commercial control available on the market. It was the only group on which the effects on the microhardness and roughness were not different from the negative control (Table 4). When mixed with the peroxide, the non‐ionic silicon dioxide particles are attracted and bonded by Van der Waals forces, creating aggregates similar to chains, with a three‐dimensional structure that immobilizes the solvent and forms the gel [33]. This gel showed the smallest reduction in hydrogen peroxide concentration, with a drop of only 1.45% (Table 5). Surprisingly, although a pH drop was detected, it did not affect the demineralization. Maybe the absence of polymer chains to retain the ions, as in the other thickeners, may have an influence on this effect. It also showed a high bleaching effect, like Carbopol and the commercial material. The mineral addition eliminated the negative effects on the microhardness, which are correlated to the demineralization values detected by Ramam spectroscopy. In fact, the maximum addition turned the enamel harder than at the baseline, also increasing the bleaching effect (Table 3).

A previous study analyzed the addition of Ca and P to pure hydrogen peroxide solution on the demineralizing effect of the enamel [11]. It was tested the addition considering the saturation concentration, with groups 50% undersaturated and others 100% saturated in relation to hydroxyapatite, as well as the maximum addition according to the solubility limit of the salts. No differences were detected between the 100% saturated in relation to the maximum addition.

In this current study, with actual bleaching gels containing different thickeners, the mineral supplementation was capable of reducing the negative effects on the tooth structure. However, the impact on the supplementation level varies among the groups. The maximum supplementation up to the solubility limit produced smaller negative effects than positive and commercial control groups for some thickeners, but the maximum addition produced significantly smaller effects for all gels tested in relation to those controls (Tables 3 and 4).

The saturation concentration values presented on Table 2 should be enough to completely prevent the negative effects, but it seems that other factors play a role or that the salts used for supplementation did not allow the Ca and P ions to be available to interact with the enamel. Another aspect is that the counterions released for the calcium acetate and sodium phosphate dibasic dodecahydrate may have interfered with the ionic equilibrium. They were chosen only by their capacity to dissolve into peroxide, without considering other implications. Maybe other salts would produce other effects. When the maximum supplementation was tested, the effect should have been magnified, but it was not significant. It is important to consider the impact of the different thickeners' ultrastructure on the interaction with the mineral ions added, and the ionic exchange with the enamel structure and hydroxyapatite crystals. The simple silicon dioxide particles ultrastructure of Aerosil 200 thickener may be the responsible for the maximum effectiveness of the mineral supplementation.

Although this study showed new information about the effects of the bleaching gels with mineral supplementation, the in vitro analysis fails to properly simulate the oral environment and the effect of the natural saliva on enamel remineralization. This way, in situ studies and finally clinical trials are necessary to confirm or deny the results. In addition, although bovine enamel is frequently used and accepted as an adequate substitute for human enamel [34], being valid in terms of comparison among groups, the full magnitude of the variables tested over the natural human enamel should be additionally determined. Future studies should also evaluate the bioavailability of the ions inside each gel formulation, since their presence inside the material seems to not guarantee the desired effects.

## 5 Conclusions

Based on the findings of this study, it was concluded that:

- The type of thickener used to produce the bleaching gel significantly influences its effects on the dental enamel;
- Mineral supplementation may reduce the deleterious effects of bleaching gels on enamel microhardness and surface roughness, but does not significantly affect the color change;
- The amount of mineral supplementation required to produce significant effects varies according to thickener used in the gel formulation;
- The bleaching gel prepared with Aerosil 200 with mineral supplementation can completely prevent the negative effects of bleaching on the dental enamel.

## Author Contributions

**Adrielle Caroline Moreira Andrade:** conceptualization, data curation, formal analysis, funding acquisition, investigation, methodology, project administration, resources, software, supervision, validation, visualization, roles/writing – original draft, writing – review and editing. **Allegra Comba:** resources, writing – review and editing. **Nicola Scotti:** resources, writing – review and editing. **Leila Es Sebar:** methodology, software, validation, visualization, writing – review and editing. **Carlos Rocha Gomes Torres:** conceptualization, data curation, formal analysis, funding acquisition, investigation, methodology, project administration, resources, software, supervision, validation, visualization, roles/writing – original draft, writing – review and editing.

## Conflicts of Interest

The authors declare no conflicts of interest.

## Data Availability

Data sharing is not applicable to this article as no new data were created or analyzed in this study.
